# Comparative Analysis of In Vitro Models to Study Antibody-Dependent Enhancement of Zika Virus Infection

**DOI:** 10.3390/v14122776

**Published:** 2022-12-13

**Authors:** Thomas Langerak, Noreen Mumtaz, Marion Koopmans, Sam Schoenmakers, Barry Rockx

**Affiliations:** 1 Department of Viroscience, Erasmus University Medical Center, 3015 CN Rotterdam, The Netherlands; 2 Division of Obstetrics and Prenatal Diagnosis, Erasmus University Medical Center, 3015 CN Rotterdam, The Netherlands

**Keywords:** Zika virus, antibody-dependent enhancement, pregnancy, dengue virus, myeloid cells

## Abstract

During the 2015–2016 outbreak of Zika virus (ZIKV) in the Americas, a previously unknown severe complication of ZIKV infection during pregnancy resulting in birth defects was reported. Since the ZIKV outbreak occurred in regions that were highly endemic for the related dengue virus (DENV), it was speculated that antibody-dependent enhancement (ADE) of a ZIKV infection, caused by the presence of cross-reactive DENV antibodies, could contribute to ZIKV disease severity. Emerging evidence indicates that, while in vitro models can show ADE of ZIKV infection, ADE does not seem to contribute to congenital ZIKV disease severity in humans. However, the role of ADE of ZIKV infection during pregnancy and in vertical ZIKV transmission is not well studied. In this study, we hypothesized that pregnancy may affect the ability of myeloid cells to become infected with ZIKV, potentially through ADE. We first systematically assessed which cell lines and primary cells can be used to study ZIKV ADE in vitro, and we compared the difference in outcomes of (ADE) infection experiments between these cells. Subsequently, we tested the hypothesis that pregnancy may affect the ability of myeloid cells to become infected through ADE, by performing ZIKV ADE assays with primary cells isolated from blood of pregnant women from different trimesters and from age-matched non-pregnant women. We found that ADE of ZIKV infection can be induced in myeloid cell lines U937, THP-1, and K562 as well as in monocyte-derived macrophages from healthy donors. There was no difference in permissiveness for ZIKV infection or ADE potential of ZIKV infection in primary cells of pregnant women compared to non-pregnant women. In conclusion, no increased permissiveness for ZIKV infection and ADE of ZIKV infection was found using in vitro models of primary myeloid cells from pregnant women compared to age-matched non-pregnant women.

## 1. Introduction

Zika virus (ZIKV) is a mosquito-borne flavivirus that can infect humans, often resulting in an asymptomatic or self-limiting mild infection. However, a ZIKV infection during pregnancy can result in severe congenital birth defects such as microcephaly, arthrogryposis, and hypertonia [1,2]. Furthermore, ZIKV infections can sporadically trigger Guillain–Barr*é* syndrome [3,4,5]. ZIKV is closely related to dengue virus (DENV) which also is a mosquito-borne flavivirus that can cause dengue hemorrhagic syndrome and dengue shock syndrome [6]. The severe presentation of a DENV infection occurs more often during a secondary DENV infection with a different serotype due to antibody-dependent enhancement (ADE) [7,8,9]. ADE is a paradoxical phenomenon in which antibodies, that normally act against pathogens and aid the immune response, can actually worsen an infection [10]. In the case of DENV, antibodies against one serotype of DENV that can cross-react with, but not cross-neutralize, another DENV serotype can help the virus to enter phagocytic cells through Fcγ-receptor (FcγR) mediated uptake, after which the virus can replicate in these cells. This results in an increased number of infected cells (extrinsic ADE) and can also lead to increased virus production per infected cell due to a shift in immune response towards a more pro-viral state (intrinsic ADE) [11,12,13]. Antigenic relatedness between DENV and ZIKV results in antibody cross-reactivity between these viruses [14,15]. Because of antibody cross-reactivity and the occurrence of the 2015–2016 outbreak of ZIKV in DENV (hyper)endemic regions, ADE of ZIKV infection was suggested as a possible mechanism behind the increased observations of ZIKV-related congenital complications during this outbreak [16]]. While there are indications from animal studies that ADE of ZIKV infection can increase the risk of transplacental ZIKV transmission, this has not been studied in detail for human pregnancies [17,18].

Multiple experimental studies investigated whether cross-reactive DENV antibodies can cause ADE of ZIKV infection in FcγR-bearing cells and in animal models [19]. Although in vitro ADE of ZIKV infection has been observed in many of these studies, this has not been observed in several animal models and in clinical studies [19,20,21]. Therefore, it seems that the result from in vitro ADE studies poorly predicts the occurrence of in vivo ADE of disease. Studying ADE in humans is challenging as it requires sufficiently powered standardized prospective cohorts which is difficult for an infection that is mostly associated with asymptomatic or mild disease. Furthermore, the methodological differences in the in vitro ADE studies make it difficult to compare the results between these studies and the conflicting results might be in part attributed to the lack of standardization of in vitro ZIKV ADE assays.

Therefore, to develop a standardized in vitro model to study ADE of ZIKV infection, we compare different cell lines and primary cell types and read-outs of in vitro ZIKV ADE assays to assess permissiveness for and immunological responses to ZIKV infection with and without the presence of cross-reactive antibodies.

Because the most severe complications of a ZIKV infection can occur during pregnancy, and the surface FcγR expression on myeloid cells is progressively upregulated during pregnancy, we then tested if there is a difference of permissiveness for ADE of ZIKV infection in myeloid cells from pregnant women compared to non-pregnant women [22].

The results of this study can ultimately provide a comparative understanding of various in vitro models employed to study ADE of ZIKV infection and gain insight into the risks of ZIKV infections during pregnancy in women who were previously exposed to other flaviviruses.

## 2. Methods

### 2.1. Human Subjects

To assess the permissiveness of primary myeloid cells for ZIKV infection and ADE of ZIKV infection, buffy coats from three healthy blood donors were obtained from the blood bank of the Netherlands (Sanquin, the Netherlands). To investigate ADE in primary cells derived from pregnant women, whole blood was collected from pregnant women from different trimesters at the outpatient clinic of the department of Obstetrics and Gynaecology at Erasmus Medical Center, Rotterdam, the Netherlands after written informed consent was obtained.

Exclusion criteria for participation were current or recent (<1 month) use of immune suppressive medication, a current or recent (<1 week) infection or vaccination, and a known immunodeficiency. For the control group, aged-matched, non-pregnant women who had not been pregnant in the 6 months prior to study participation were recruited. Exclusion criteria for the non-pregnant women were the same as for the pregnant women. Approval for this study was granted by the Medical Ethical Committee of Erasmus Medical Center (MEC-2021-0134).

### 2.2. Cell Lines

Cell lines tested to study ADE were selected based on previous studies regarding ADE of ZIKV and DENV infection [23,24,25,26,27]. K562 cells were obtained from ATCC (CCL-243) and were cultured in Iscove’s Modified Dulbecco’s Medium (IMDM; Lonza, Basel, Switzerland) supplemented with 10% fetal bovine serum (FBS, Sigma-Aldrich, St. Louis, MO, USA), 100 U/mL penicillin, and 100 μg/mL streptomycin (Lonza) at 37 °C, 5% CO_2_. U937 and THP-1 cells (kindly provided by the department of Immunology of Erasmus Medical Center) were cultured in RPMI 1640 (Lonza) supplemented with 10% FBS, 100 U/mL penicillin, and 100 μg/mL streptomycin at 37 °C, 5% CO_2_. Vero cells (African green monkey kidney epithelial cells, ATCC CCL-81) were cultured in Dulbecco’s modified Eagle’s medium (DMEM, Lonza) with 10% FBS, 100 U/mL penicillin, 100 μg/mL streptomycin, and 2 mM glutamine. All cell lines were tested and found to be mycoplasma free.

### 2.3. Primary Human Cells

For isolation of peripheral blood mononuclear cells (PBMCs), blood was layered on Ficoll-Paque PLUS density gradient media (GE Healthcare Life, Singapore) and centrifuged for 20 min at 800× *g* without brake. After centrifugation, the PBMC layer was isolated, washed three times with PBS, and frozen at −135 °C until further use. Monocytes were isolated from frozen PBMCs using magnetic-based CD14-positive selection according to the manufacturer’s instructions and the Pan Monocyte Isolation Kit (both Miltenyi Biotec). The purity of the monocytes, based on CD14 positivity, was 87.6% as determined with flow-cytometry (Figure S1). To differentiate monocytes into macrophages and dendritic cells, monocytes were plated at a density of 100,000 cells per well in 96-well plates in complete RPMI 1640 (Lonza) medium supplemented with 4200 IU/mL macrophage colony stimulating factor (M-CSF; R&D systems, Minneapolis, MI, US) for macrophages, and with 1000 IU/mL granulocyte macrophage colony stimulating factor (GM-CSF; R&D systems, US) and 500 IU/mL IL-4 for dendritic cells, for six days. For maturation of immature dendritic cells, lipopolysaccharides (Sigma-Aldrich) were added to the cells on day six for 48 h in a concentration of 1 µg/mL. The maturation of dendritic cells was confirmed by upregulated expression of maturation markers, including CD80 and CD83, compared to immature dendritic cells (Figure S2).

### 2.4. Virus and Virus Quantification

An Asian lineage ZIKV strain was used for all experiments (Suriname ZIKVNL00013, EVAg no. 011V-01621). The virus was grown in Vero cells and passage number 3 was used for the current study. Viral titers in supernatants, expressed as a 50% tissue culture infective dose (TCID_50_), was determined by 10-fold dilution endpoint titration on Vero cells and calculated with the method of Kärber [28].

### 2.5. ZIKV Infection

For infection experiments, cells were seeded in 96-well plates at a density of 100,000 cells per well and infected with ZIKV at a multiplicity of infection (MOI) of 0.5, 1, 5, or 10 for 1 hour at 37 °C, 5% CO_2_. After incubation, the supernatant was removed, and cells were washed three times. Cell supernatants were collected at day 0 and day 2 post infection. The supernatant was stored at −80 °C until further use. Experiments were performed in triplicates.

### 2.6. Antibody-Dependent Enhancement Assay

For ADE assays, ZIKV (MOI 0.5) was incubated with different concentrations of the humanized IgG1 pan-flavivirus antibody 4G2 (hu4G2, Native Antigen Company, Kidlington, UK) at 37 °C to form immune complexes. After an hour of incubation, the virus-antibody mixture was added to the cells in 96-well plates and incubated for one hour at 37 °C, 5% CO_2_. Subsequently, cells were washed three times and the supernatant was collected on day 2 post infection and frozen at −80 °C until further use. For FcγR blocking experiments, cells were pre-incubated for one hour with 10 μg/mL FcγRI-, FcγRII-, and/or FcγRIII-blocking antibodies (clones 10.1, 6C4, and 3G8, respectively, all eBioscience) at 37 °C before infection with ZIKV or ZIKV + hu4G2.

### 2.7. Flow Cytometry

In some ADE experiments, flow-cytometry was used as a read-out to determine the percentage of infected cells. For this, cells were collected on day two post infection and stained with live/dead stain. Cells were blocked with 10% normal goat serum (NGS, Dako, Glostrup, Denmark) and total human Fc block (BD Biosciences). After fixation and permeabilization using BD Cytofix/Cytoperm (BD Biosciences, Franklin Lakes, NJ, USA), intracellular staining for ZIKV E-protein was performed with a mouse 4G2 antibody (MAB10216, clone D1-4G2-4-15; Millipore, Darmstadt, Germany) followed by an APC/Cy-7 conjugated goat anti-mouse IgG2a secondary antibody (Abcam, Cambridge, UK). Flow cytometry was performed with the FACS Lyric machine (BD Biosciences, USA). Data were analyzed using FlowJo 10.6.1 software (Ashland, OR, USA). All experiments were performed three times (biological replicates), and each experiment included duplicate (technical replicates) measurements from which the average was calculated and used for further analysis. To demonstrate that intracellular ZIKV-E staining represents productive infection and not just phagocytosis, some ZIKV-infected cells were also stained with an anti-DENV NS3 antibody (E1D8, My Biosource, San Diego, CA, USA), which has been shown to cross-react with ZIKV NS3 [29].

### 2.8. Cytokine Detection in Supernatant

Cytokines were detected in the supernatant of infected cells using a 13-plex bead-based fluorescence assay according to the manufacturer’s instruction (LEGEND-plex Human Anti-Virus Response Panel, Biolegend). Briefly, beads conjugated with a specific antibody against cytokines were incubated with the cell supernatant of either uninfected cells, ZIKV-infected cells, or ZIKV + hu4G2 infected cells. Subsequently, a detection antibody cocktail was added, and a read-out of the assay was performed with flow cytometry (FACS lyric, BD Biosciences, USA). The cytokines that could be detected with this assay were IL-1β, IL-6, IL-8, IL-10, IL-12p70, IFN-α, IFN-β, IFN-λ1, IFN-λ2/3, IFN-γ, TNF-α, IP-10, and GM-CSF. Data were analyzed with LEGEND-plex data analysis software (Biolegend).

### 2.9. Statistical Analysis

Statistical analyses were performed with GraphPad Prism 8. Significant differences between groups in the ADE assays were determined with either a Students’ t-test or ANOVA, with Dunnett’s post-hoc test or for non-normally distributed data a Mann-Whitney U test or Kruskal–Wallis tests, followed by Dunn’s post-hoc test. Correlations between viral titers and the percentage of infected cells were determined with Spearman’s rank correlation coefficient. Within the different pregnancy trimester groups, paired analysis between conditions with and without ADE was performed with a paired Mann-Whitney U test. A *p*-value ≤ 0.05 was considered a statistically significant difference.

## 3. Results

### 3.1. Permissiveness for ZIKV Infection

**Figure 1:**
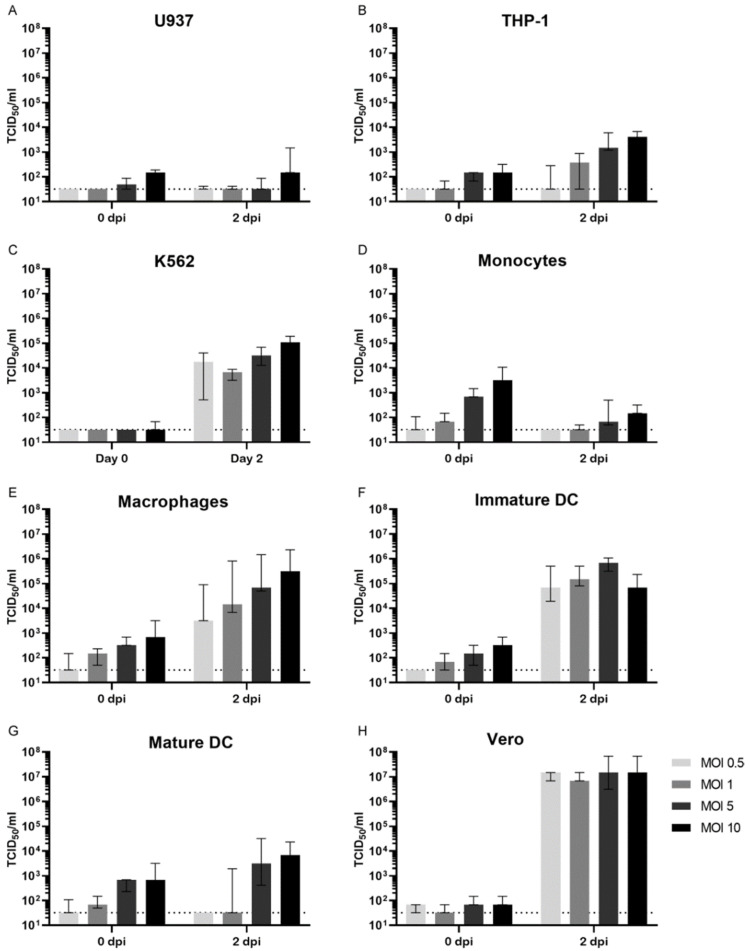
ZIKV infection of FcγR-bearing cell lines and Vero cells. Myeloid cell lines (**A**–**C**), primary myeloid cells (**D**–**G**), and Vero cells (**H**) were infected with ZIKV at different MOI’s for 48 h after which ZIKV titers were determined in supernatants. Dotted lines represent the lower limit of detection. Bars represent median ZIKV titer ± interquartile range. DC; dendritic cells.

We first studied the permissiveness for ZIKV infection of cells that are commonly used for in vitro ZIKV ADE assays; the cell lines U937 [14,16], K562 [26,30] and THP-1 [31,32] as well as primary monocytes [33], monocyte-derived macrophages, monocyte-derived immature dendritic cells (immature DCs) and monocyte derived mature dendritic cells (mature DCs) [34,35]. Cells were infected with ZIKV at varying multiplicities of infection (MOI) for 48 h after which viral titers were determined in the supernatants. Infection of U937 cells with ZIKV with an MOI of 0.5, 1, or 5 did not result in viral replication while infection with ZIKV at an MOI of 10 resulted in low titers (147 TCID_50_/mL, Figure 1A). THP-1 cells and especially K562 cells were more permissive for ZIKV infection (peak titer 4.1 × 10^3^ and 1.1 × 10^5^ TCID_50_/mL, respectively, Figure 1B,C). For the primary cells, replication with low titers was observed in monocytes (peak titer 147 TCID_50_/mL, Figure 1D), while monocyte-derived macrophages and immature DCs were permissive to ZIKV infection resulting in high ZIKV titers in supernatants (peak titers 3.2 × 10^5^ and 6.8 × 10^5^ TCID_50_/mL, respectively, Figure 1E,F). Mature DCs were less permissive than immature DCs and could only be infected with a high MOI of ZIKV (peak titer 6.8 × 10^3^ TCID_50_/mL, Figure 1G). Vero cells were included as a reference since these cells are known to be highly permissive for ZIKV infection. As expected, peak viral titers in supernatants of infected Vero cells were high, regardless of the MOI of ZIKV (peak titer 1.5 × 10^7^ TCID_50_/mL, Figure 1H).

### 3.2. ADE of ZIKV Infection in Myeloid Cell Lines and Primary Myeloid Cells

To determine the potential for ADE of ZIKV infection of the above-mentioned cell lines and primary cells, we subsequently performed ADE assays with these cells. Cells were infected with ZIKV alone or ZIKV that was pre-incubated with increasing concentrations of a humanized pan-flavivirus monoclonal antibody (hu4G2) at the lowest MOI (0.5) that was used in the permissiveness experiments in Figure 1. After 48-h, two commonly used read-outs for ADE assays were used; virus titration of supernatants and flow cytometry analysis to determine the percentage of infected cells.

**Figure 2:**
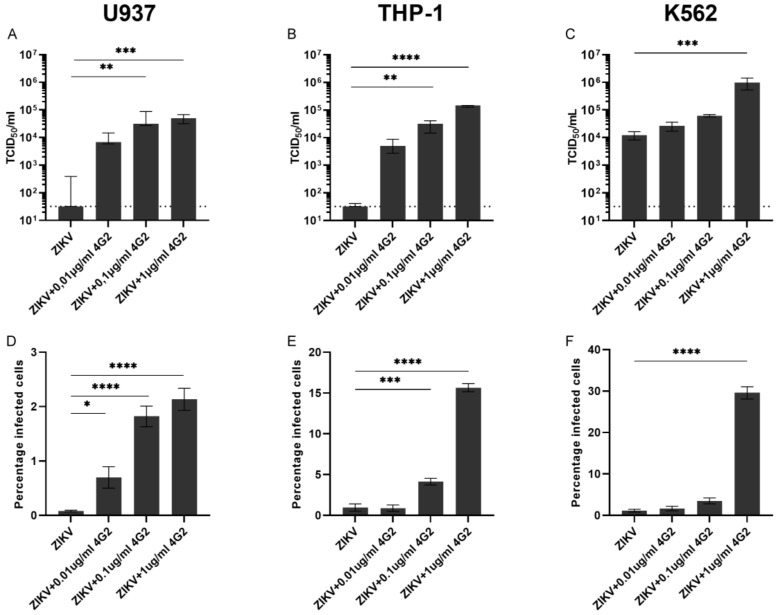
ADE of ZIKV infection in U937, THP-1, and K562 cells. Cells were infected for 48 h with ZIKV at an MOI of 0.5 with and without increasing concentrations of the pan-flavivirus humanized monoclonal antibody 4G2 (hu4G2). ZIKV titers were determined in supernatants (**A**–**C**) and the percentage of infected cells was determined with flow cytometry (**D**–**F**). Bars represent median titer ± IQR (**A**–**C**) and mean percentage of infected cells ± SEM (**D**–**F**). Statistical significance was determined with the Kruskal–Wallis test with Dunn’s post hoc test or a one-way ANOVA with Dunnett’s post hoc test, comparing the conditions with hu4G2 to infection with only ZIKV. * *p* < 0.05, ** *p* < 0.01, *** *p* < 0.001, ****p < 0.0001.

Pre-incubation of ZIKV with hu4G2 resulted in a concentration-dependent increase of infectious titer and percentage of infected cells in all three tested cell lines (Figure 2). Highest viral titers and the percentage of infected cells were observed in K562 cells (9.7 × 10^5^ TCID_50_/mL and 29.6% infected cells) and lowest in U937 cells (5.0 × 10^4^ TCID_50_/mL and 2.1% infected cells) after infection with ZIKV + 1µg/mL hu4G2. For all cell lines, there was a strong correlation between the viral titers in supernatants and the percentage of infected cells (r = 0.85 for U937, r = 0.85 for THP-1 and r = 0.82 for K562, *p* < 0.0001 for all, Figure S3A–C).

**Figure 3:**
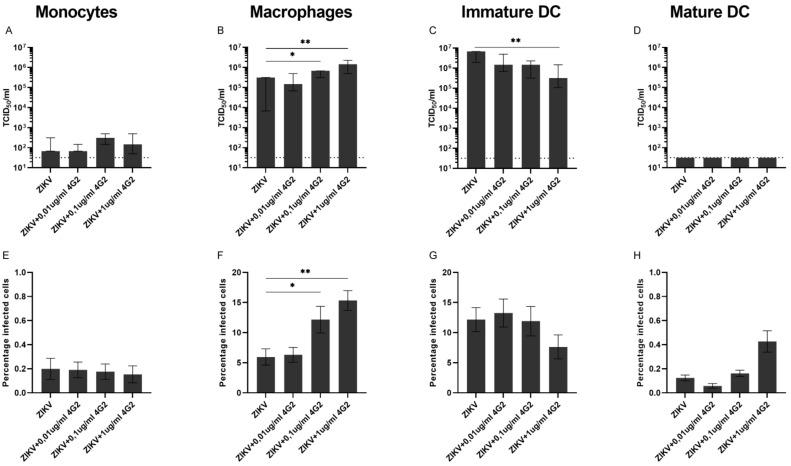
ADE of ZIKV infection in primary myeloid cells. FcγR-bearing human myeloid cells were infected for 48 h with ZIKV at an MOI of 0.5 with and without increasing concentrations of the pan-flavivirus humanized monoclonal antibody 4G2 (hu4G2). Viral titers in supernatants were determined with titration (**A**–**D**) and the percentage of infected cells was determined with flow cytometry (**E**–**H**). Bars represent median titer ± IQR (**A**–**D**) and mean a percentage of infected cells ± SEM (**E**–**H**). Statistical significance was determined with the Kruskal–Wallis test with Dunn’s post hoc test or a one-way ANOVA with Dunnett’s post hoc test, comparing the conditions with hu4G2 to infection with only ZIKV. * *p* < 0.05, ** *p* < 0.01. N = 3 donors.

For the primary cells, ADE of ZIKV infection was not observed in monocytes nor in mature DCs, while for immature DCs, the addition of hu4G2 even partially inhibited ZIKV infection (Figure 3C,G). We ruled out that a CD14-positive selection of monocytes influences the permissiveness of these cells for ZIKV infection due to stimulation of CD14 by using a non-CD14-based negative selection kit to isolate monocytes from PBMCs. The permissiveness for ZIKV infection was comparable between the negatively and positively isolated monocytes when infected with ZIKV at a MOI of 0.5, 1, and 5 and slightly increased when infected with an MOI of 10 (2315 vs. 147 TCID_50_/mL *p* = 0.002, Figure S6).

In monocyte-derived macrophages, ADE of ZIKV infection was dependent on the concentration of hu4G2 as shown by an increase in viral titers in supernatants and an increase in the percentage of infected cells (1.5 × 10^6^ TCID_50_/mL and 15.3% infected cells with 1 µg/mL hu4G2, *p* < 0.01, Figure 3B,F). Similar to the cell lines, a strong positive correlation was found in monocyte-derived macrophages between the viral titers in supernatants and the percentage of infected cells (r = 0.82, *p* < 0.0001, Figure S3D).

Collectively, these results demonstrate that ADE of ZIKV infection can be observed in the myeloid cell lines U937, K562, and THP-1. For primary cells, ADE of ZIKV infection is only observed in monocyte-derived macrophages.

### 3.3. Fcγ Receptors and ADE of ZIKV Infection

One of the main relevant differences between the cell lines used for ADE assays in this study is the expression of Fcγ-receptors (FcγRs). U937 and THP-1 cells express FcγRI and –II (CD64 and CD32) while K562 cells only express FcγRII (Figure S4) [36]. For primary myeloid cells, FcγRI and –II are expressed by monocytes, monocyte-derived macrophages, and can be induced in dendritic cells, while FcγRIII (CD16) is expressed by monocyte-derived macrophages and dendritic cells and on intermediate and non-classical monocytes [37,38,39,40]. In the cells permissive for ADE of ZIKV infection, we tested which FcγRs were important for this ADE. Therefore, we performed ZIKV ADE assays with the highest tested concentration of hu4G2 (1 µg/mL) and pre-incubated the cells with monoclonal antibodies against FcγRI, FcγRII, FcγRIII, or with a blocker of all Fc-receptors (total Fc Block) to block the interaction of hu4G2 with these FcγRs.

**Figure 4:**
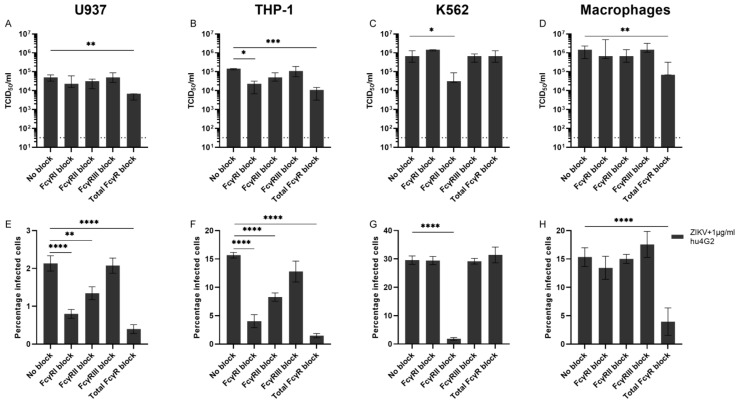
FcγRI and FcγRII contribute to ADE of ZIKV infection in FcγR cell lines and primary cells. FcγR-bearing human cell lines and myeloid cells that are permissive to ADE of ZIKV infection were pre-incubated with monoclonal antibodies against FcγRs or with Human BD Fc Block before adding ZIKV (MOI 0.5) that was pre-incubated with 1 µg/mL of the pan-flavivirus humanized monoclonal antibody 4G2 (hu4G2). Viral titers in supernatants were determined with titration (**A**–**D**) and the percentage of infected cells was determined with flow cytometry (**E**–**H**). Bars represent median titer ± IQR (**A**–**D**) and mean percentage of infected cells ± SEM (**E**–**H**). Statistical significance was determined with the Kruskal–Wallis test with Dunn’s post hoc test or a one-way ANOVA with Dunnett’s post hoc test, comparing the conditions with hu4G2 to infection with only ZIKV. * *p* < 0.05, ** *p* < 0.01, *** *p* < 0.001, ****p < 0.0001. N = 3 donors for monocyte-derived macrophages.

ADE of ZIKV infection in U937 and THP-1 cells was mainly inhibited by blocking FcγRI and by adding total Human Fc Block and to a lesser extent by blocking FcγRII while blocking. FcγRIII did not reduce viral titers or percentage of infected cells (Figure 4A,B,E,F). For K562, which only expresses FcγRII, ADE of ZIKV infection was significantly inhibited by blocking FcγRII but not by the other FcγR blockers (Figure 4C,G). For macrophages, blocking FcγRI and FcγRII resulted in a slight, but not statistically significant, decrease in the percentage of infected cells, while adding total Fc Block did significantly reduce ADE of ZIKV infection in these cells (Figure 4D,H). Collectively, these data demonstrate that ADE of ZIKV infection, induced by a monoclonal antibody, can be inhibited by blocking FcγRI (except for K562) and to a lesser extent FcγRII.

### 3.4. Cytokine Quantification

ADE is believed to be one of the main mechanisms responsible for a cytokine storm that occurs during severe DENV infections; therefore, we determined cytokine production of all cells during ADE of ZIKV infection [41].

**Figure 5:**
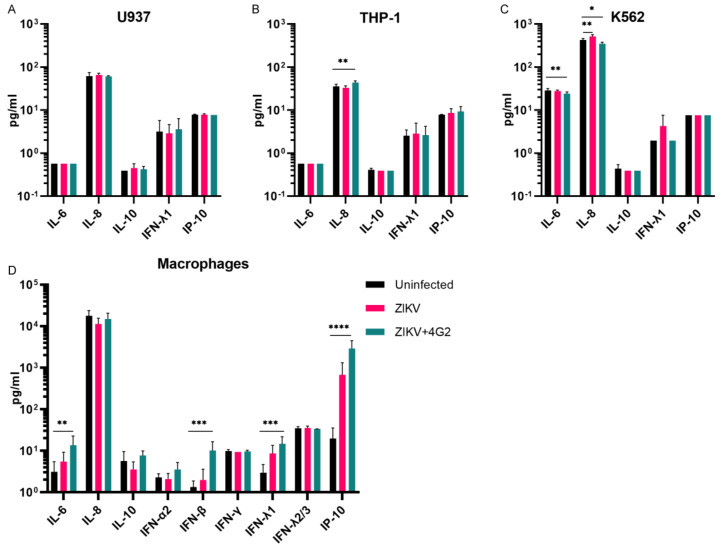
A modest increase of cytokine concentrations in supernatants of FcγR-bearing cells during ADE of ZIKV infection. A panel of 13 cytokines were determined in U937 cells (**A**), THP-1 cells (**B**), K562 cells (**C**) and monocyte-derived macrophages (**D**) 48 h after mock infection or infection with ZIKV or ZIKV that was pre-incubated with 1 µg/mL of the pan-flavivirus humanized monoclonal antibody 4G2 (hu4G2). Only the cytokines that could be detected in the supernatants of the cells are illustrated in the graphs. Bars represent mean ± SEM. Statistical significance was determined with a one-way ANOVA with Dunnett’s post hoc test, comparing uninfected cells with ZIKV infected and ZIKV + hu4G2 infected. * *p* < 0.05, ** *p* < 0.01, *** *p* < 0.001, **** *p* < 0.0001.

Cytokines were determined in supernatants of cells that were permissive for ZIKV ADE, 48 h after either mock infection, ZIKV infection, or ADE of ZIKV infection. Only a limited number of cytokines could be detected in the supernatants of U937, K562, and THP-1 cells and differences between cytokine concentrations of uninfected cells compared to cells infected with ZIKV or ZIKV + hu4G2 were small (Figure 5A–C). For THP-1 cells, IL-8 production of cells infected with ZIKV + 4G2 was statistically significantly higher compared to uninfected cells (average 43.7 vs. 35.7 pg/mL, *p* = 0.007), while for K562 cells, a decrease in IL-8 production was found during ADE of ZIKV infection compared to uninfected cells (426.4 vs. 350.8 pg/mL, *p* = 0.014). IL-6 production was lower in K562 infected with ZIKV + 4G2 compared to uninfected K562 cells (24.2 vs. 28.8 pg/mL, *p* = 0.005). Compared to the cell lines, more cytokines could be detected in supernatants of monocyte-derived macrophages (Figure 5D). In line with a previous observation, there was an increased production of IL-6 in monocyte-derived macrophages during ADE of ZIKV infection compared to mock infection (13.6 vs. 3.1 pg/mL, *p* = 0.003, Figure 5D) [35]. Furthermore, production of IFN-β, IFN-λ1, and IP-10 was significantly increased during ADE of ZIKV infection compared to uninfected cells (10.1 vs. 1.3 pg/mL for IFN-β, 14.7 vs. 3.0 pg/mL for IFN-λ1, and 2889 vs. 19.8 pg/mL for IP-10, *p* < 0.001, *p* < 0.001, and *p* < 0.0001, respectively, Figure 5D). An increase in IL-10 production during ADE of infection, which is thought to play an important role in intrinsic ADE of DENV infections, was not observed in any of the cells.

### 3.5. ZIKV Permissiveness in Myeloid Cells from Pregnant Women

Due to the potential detrimental effects of a ZIKV infection during pregnancy, we wanted to test whether FcγR-bearing primary myeloid cells, collected during different trimesters of pregnancy, are more permissive for ZIKV infection and ADE of ZIKV infection compared to non-pregnant women. Therefore, we isolated PBMCs from 30 pregnant women during each of the three trimesters, as well as from 10 non-pregnant women. Based on the above presented results, we decided to use monocyte-derived macrophages to test potential differences in the permissiveness of (ADE of) ZIKV infection. As we found a strong positive correlation between infection titers and percentage of infected cells, we decided to continue with virus titration as a read-out for (ADE of) ZIKV infection in these experiments. With this read-out, an increase in infected cells (extrinsic ADE) as well as an increase of viral production per infected cell (intrinsic ADE) can be detected, which is not the case when determining the percentage of infected cells with flow cytometry. Even though in monocytes of three healthy donors, we only found low-grade ZIKV infection and no ADE (Figure 1D and Figure 3A,E), we also infected monocytes of the pregnant and non-pregnant women with ZIKV because a higher susceptibility of monocytes for ZIKV infection has been observed during the first trimester of pregnancy [42].

**Figure 6:**
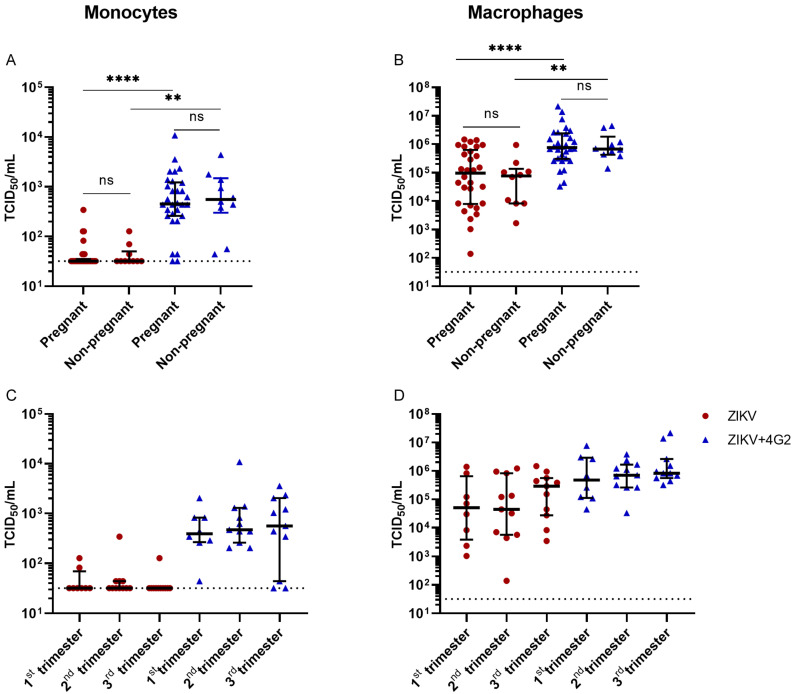
No differences in permissiveness for ZIKV infection and ADE of ZIKV infection in monocytes (**A**) or monocyte-derived macrophages (**B**) from pregnant women compared to non-pregnant women. ZIKV titers did not differ after ZIKV infection of ADE of ZIKV infection in monocytes (**C**) and monocyte-derived macrophages (**D**) collected from pregnant women in different trimesters of pregnancy. Cells were infected for 48 h with ZIKV at an MOI of 0.5 with and without 1 µg/mL pan-flavivirus humanized monoclonal antibody 4G2 (4G2) and viral titers in supernatants were determined by titration. Bars represent median titer ± IQR. Statistical significance was determined with the Kruskal–Wallis test with Dunn’s post hoc test ** *p* < 0.01, **** *p* < 0.0001.

No difference was found in permissiveness for ZIKV infection in monocytes or macrophages over the different trimesters of pregnancy nor between pregnant women compared to non-pregnant women (Figure 6B and Figure S5). ADE of ZIKV infection was observed in monocytes and monocyte-derived macrophages from pregnant women and non-pregnant women. There was no difference in peak ADE titers in supernatants of primary cells from pregnant women, independent of gestational age, compared to non-pregnant women (Figure 6A,B). Furthermore, no difference was found in ZIKV permissiveness and ADE potential between monocytes and macrophages from women at different gestational ages at the time of sample collection (first, second, or third trimester) (Figure 6D).

Collectively, these data demonstrate that no enhanced susceptibility of ZIKV infection and ADE of infection is present in monocytes and monocyte-derived macrophages collected from pregnant women compared to non-pregnant women.

## 4. Discussion

In this paper, we found that the myeloid cell lines U937, K562, and THP-1 were all permissive for ADE of ZIKV infection, as has been shown by others [16,31,43]. The highest ZIKV titers were obtained in K562 cells, followed by THP-1 and U937 cells, the latter of which was non-permissive to ZIKV infection in the absence of cross-reactive antibodies. Blocking of FcγRI significantly reduced ADE of ZIKV infection in both U937 and THP-1 cells which, for U937 cells, is in line with findings for DENV ADE [43,44,45]. However, contrary to our observations, FcγRIIa has been reported to be the main FcγR responsible for ADE of DENV and ZIKV infections [33,40,46]. In these previous studies, ADE of DENV or ZIKV infection was mainly induced by convalescent polyclonal sera while in this study, we only use a humanized IgG1 monoclonal antibody (hu4G2). A higher affinity of hu4G2 for FcγRI compared to FcγRII could possibly explain why ADE of ZIKV in these experiments mainly depend on FcγRI.

To relate the findings from in vitro cell line models to potential primary cell targets, we evaluated the permissiveness for (ADE) of ZIKV infection in primary human myeloid cells, which are generally considered to be more representative of the in vivo human situation compared to cell lines. Of the primary myeloid cells isolated from healthy blood donors, only monocyte-derived macrophages and immature dendritic cells were highly permissive to ZIKV infection, while ADE of ZIKV infection was only observed in monocyte-derived macrophages. A high permissiveness of monocyte-derived macrophages for ZIKV infection and ADE of ZIKV infection has been previously observed [27]. The high permissiveness of immature dendritic cells for ZIKV infection but not for ADE of ZIKV infection has been previously observed for both DENV and ZIKV and might be caused by a high expression of the flavivirus entry cofactor DC-SIGN by these cells, which has shown to be inversely correlated to the rate of ADE of infection [33,47,48]. Our finding is in line with the previously published study by Li et al., 2018 [33], while few studies have shown the conflicting observation about the susceptibility of mature DCs to ZIKV infection [49,50]. These conflicting findings could be explained based on a previously published study for DENV [51], where it was shown that DENV infection of monocytes/macrophages (MO/Mφ) was suppressed due to LPS treatment via a CD14-dependent mechanism of monocytes. This could possibly explain our current observation regarding the non-permissiveness of mature DCs to ZIKV infection. In the monocyte-derived macrophages, ADE of ZIKV infection seemed to be dependent on both FcγRI and FcγRII since the blocking of these receptors individually resulted in a non-statistically significant reduction of ADE of infection, while blocking all FcγRs with total Fc Block did result in a significant reduction of ADE. The low permissiveness of monocytes for ZIKV infection that we found in this study is in contrast with results from other studies that showed that monocytes are the main targets of ZIKV infection in blood [29,33,42,52]. One possible explanation for this can be that there is a variation in the tropism for monocytes between the different Asian lineage ZIKV isolates used in the previous studies and this study [53].

The main difference in cytokine production of the cell lines upon (ADE of) ZIKV infection was observed for IL-8, which was induced in THP-1 cells while reduced in K562 cells. IL-8 is a chemoattractant for neutrophils which are important effector cells of the innate immune system. A reduction of IL-8 production during ADE of ZIKV infection could reflect a dampened innate antiviral immune response as is observed during ADE of ZIKV infection in fetal macrophages [54,55]. The induction of IFN-β, IFNλ1, and IP-10 in monocyte-derived macrophages during ZIKV ADE is in line with a pro-inflammatory immune response while an induction of IL-6 can indicate a T-helper cell 2 biased immune response as is seen during intrinsic ADE [54,56,57]. However, as with the cell lines, we did not detect an increase in IL-10 production in monocyte-derived macrophages during ADE of ZIKV infection, which has been suggested to be one of the hallmarks of intrinsic ADE for DENV and seems to contribute to disease severity in severe DENV infections [11,12,57,58,59]. It is possible that a non-increased IL-10 production during ADE of infection is specific for ZIKV as we and others have not observed increased IL-10 production during ADE of ZIKV infection in fetal macrophages and in primary human cells [35,55,60].

When combining the results regarding peak viral titer and the percentage of infected cells during ADE of ZIKV infection, THP-1 and K562 cells resemble the monocyte-derived macrophages better than U937 cells, while U937 cells resemble monocytes. Regarding FcγRs’ expression, monocyte-derived macrophages are resembled by U937 and THP-1 cells but not by K562 cells since these only express FcγRII. In general, these data suggest that the THP-1 cell line resembles monocyte-derived macrophages, which are the best for ADE of ZIKV infection concerning titers and FcγRs responsible for ADE of ZIKV infection while primary monocytes are best resembled by U937 cells. Since ADE of ZIKV infection can be induced in all three cell lines, all three of these cells can be used for in vitro screening of antibodies for ADE potential.

The role of ADE in the context of pregnancy is critical to study not only because ZIKV can cause congenital abnormalities after maternal infection and ZIKV co-circulates with DENV, but also due to physiological changes that occur during pregnancy [17,22]. Therefore, after validating the assays and read-outs using cell lines and primary cells, we determined the ADE of ZIKV infection in potential target cells derived from the pregnancy cohort. The ZIKV titers that we found in ZIKV-infected monocytes from pregnant women and non-pregnant women were in line with a previous study by Foo et al. [42]. One difference is, however, that in this previous study, a small increase in permissiveness for ZIKV infection was found in monocytes from pregnant women in the first trimester compared to non-pregnant women based on ZIKV NS1 viral load, while this is not observed in the current study [42]. This could possibly be explained by differences in the experimental set-up, e.g., infection of whole blood vs. infection of PBMCs-derived monocytes and the specific ZIKV isolate that was used. As with the monocytes, no differences were found in monocyte-derived macrophages for ZIKV infection and ADE of ZIKV infection. These results indicate that monocytes and monocyte-derived macrophages from pregnant women are not more permissive for ZIKV infection and ADE of ZIKV infection compared to non-pregnant women, independent of trimester.

Though our findings are consistent and validated at various levels, there are limitations to this study. First, to avoid the variability among the tested in vitro models because of IgG subclasses, we only used humanized 4G2 as an enhancing antibody and not, e.g., polyclonal sera containing DENV antibodies. However, it is likely that there are differences in affinity for FcγRs between monoclonal hu4G2 and polyclonal serum consisting of multiple IgG subclasses that may also have an impact on the final outcome of ADE assay [61]. Furthermore, differences in glycosylation status of this monoclonal antibody compared to polyclonal sera are also likely to influence the affinity for different FcγRs [62,63]. The exact role of interactions of immune complexes with different FcγRs on the outcome of ADE of infection and the role of glycosylation of these antibodies on this is an interesting topic that should be studied in more detail. Furthermore, we only used one MOI (0.5) and one concentration of hu4G2 for all infection experiments to limit the number of variables. It could be that with a higher MOI or with a (even) higher concentration of hu4G2, ADE of ZIKV infection would be observed in, e.g., monocytes and mature dendritic cells. However, it is not likely that these conditions will occur in vivo. Lastly, for the pregnant cohort due to limited sample availability, we tested only PBMCs-derived myeloid cells to validate ZIKV-ADE in an in vitro culture system. This does not allow us to study the possible effects of hormonal changes that occur during pregnancy on permissiveness for (ADE of) ZIKV infection, which needs further validation in a well-defined cohort.

In conclusion, we found that U937, THP-1, and K562 cell lines can be used for in vitro ADE assays for ZIKV as well as monocyte-derived macrophages. Regarding permissiveness for ZIKV infection and ADE of ZIKV infection, THP-1 cells mimic the results that are obtained with monocyte-derived macrophages, while U937 cells mimic the results of monocytes. No increased permissiveness for ZIKV infection and ADE of ZIKV infection was found in primary myeloid cells from pregnant women compared to age-matched non-pregnant women. These data are in line with data from a study with pregnant non-human primates and indicate that during pregnancy, pre-existing DENV immunity is not likely to be a risk for a more severe ZIKV infection in the mother [64]. A possible facilitating role of DENV immunity on the risk of transplacental ZIKV transmission is not ruled out based on the results of this study and is suggested in multiple experimental studies and should be studied in more detail in human studies [17,18,55,60,65].

## Data Availability

Not applicable.

## Supplementary Materials

The following supporting information can be downloaded at: https://www.mdpi.com/article/10.3390/v14122776/s1, Figure S1: CD14+ expression of monocytes that were magnetically isolated from frozen PBMCs using anti- human CD14 microbeads; Figure S2: Characterization of dendritic cell surface expression markers using flow cytometry; Figure S3: Strong correlation between viral titers in supernatants and percentage of infected cells U937 cells; Figure S4: FcγR expression in U937, THP-1 and K562 cells. Cells are illustrated in the red graphs while isotype controls are illustrated in blue; Figure S5: Paired ZIKV titers determined in supernatants of monocytes and macrophages from pregnant women (per trimester) and non-pregnant women infected with ZIKV and with ZIKV+hu4G2 to induce ADE of infection; Figure S6: Monocytes were isolated from PBMC’s with a CD14+ isolation kit (pos. selected) or with a pan monocyte isolation kit in which monocytes are negatively selected (untouched, neg. selected).

## References

[1] Mlakar J., Korva M., Tul N., Popović M., Poljšak-Prijatelj M., Mraz J., Kolenc M., Resman Rus K., Vesnaver Vipotnik T., Fabjan Vodušek V. (2016). Zika Virus Associated with Microcephaly. N. Engl. J. Med..

[2] Rasmussen S.A., Jamieson D.J., Honein M.A., Petersen L.R. (2016). Zika Virus and Birth Defects—Reviewing the Evidence for Causality. N. Engl. J. Med..

[3] Cao-Lormeau V.-M., Blake A., Mons S., Lastère S., Roche C., Vanhomwegen J., Dub T., Baudouin L., Teissier A., Larre P. (2016). Guillain-Barré Syndrome outbreak associated with Zika virus infection in French Polynesia: A case-control study. Lancet.

[4] Langerak T., van Rooij I., Doornekamp L., Chandler F., Baptista M., Yang H., Koopmans M.P.G., GeurtsvanKessel C.H., Jacobs B.C., Rockx B. (2021). Guillain-Barré Syndrome in Suriname; Clinical Presentation and Identification of Preceding Infections. Front. Neurol..

[5] Brasil P., Sequeira P.C., Freitas A.D., Zogbi H.E., Calvet G.A., de Souza R.V., Siqueira A.M., de Mendonca M.C.L., Nogueira R.M.R., de Filippis A.M.B. (2016). Guillain-Barré syndrome associated with Zika virus infection. Lancet.

[6] Guzman M.G., Gubler D.J., Izquierdo A., Martinez E., Halstead S.B. (2016). Dengue infection. Nat Rev Dis Primers..

[7] Katzelnick L.C., Gresh L., Halloran M.E., Mercado J.C., Kuan G., Gordon A., Balmaseda A., Harris E. (2017). Antibody-dependent enhancement of severe dengue disease in humans. Science.

[8] Halstead S.B. (2017). Dengvaxia sensitizes seronegatives to vaccine enhanced disease regardless of age. Vaccine.

[9] Halstead S.B., Nimmannitya S., Cohen S.N. (1970). Observations related to pathogenesis of dengue hemorrhagic fever. IV. Relation of disease severity to antibody response and virus recovered. Yale J. Biol. Med..

[10] Hawkes R.A. (1964). Enhancement of the Infectivity of Arboviruses by Specific Antisera Produced in Domestic Fowls. Aust. J. Exp. Biol. Med. Sci..

[11] Flipse J., Wilschut J., Smit J.M. (2012). Molecular mechanisms involved in antibody-dependent enhancement of dengue virus infection in humans. Traffic.

[12] Halstead S.B., Mahalingam S., Marovich M.A., Ubol S., Mosser D.M. (2010). Intrinsic antibody-dependent enhancement of microbial infection in macrophages: Disease regulation by immune complexes. Lancet Infect. Dis..

[13] Halstead S.B. (2014). Dengue Antibody-Dependent Enhancement: Knowns and Unknowns. Microbiol. Spectr..

[14] Priyamvada L., Quicke K.M., Hudson W.H., Onlamoon N., Sewatanon J., Edupuganti S., Pattanapanyasat K., Chokephaibulkit K., Mulligan M.J., Wilson P.C. (2016). Human antibody responses after dengue virus infection are highly cross-reactive to Zika virus. Proc. Natl. Acad. Sci. USA.

[15] Langerak T., Kasbergen L., Chandler F., Brinkman T., Faerber Z., Phalai K., Ulbert S., Rockstroh A., Bruin E., Koopmans M. (2021). Zika Virus Antibody Titers Three Years after Confirmed Infection. Viruses.

[16] Dejnirattisai W., Supasa P., Wongwiwat W., Rouvinski A., Barba-Spaeth G., Duangchinda T., Sakuntabhai A., Cao-Lormeau V.-M., Malasit P., Rey F.A. (2016). Dengue virus sero-cross-reactivity drives antibody-dependent enhancement of infection with zika virus. Nat. Immunol..

[17] Brown J.A., Singh G., Acklin J., Lee S., Duehr J., Chokola A., Frere J., Hoffman K.W., Foster G.A., Krysztof D. (2019). Dengue Virus Immunity Increases Zika Virus-Induced Damage during Pregnancy. Immunity.

[18] Rathore A.P.S., Saron W.A.A., Lim T., Jahan N., St. John A.L. (2019). Maternal immunity and antibodies to dengue virus promote infection and Zika virus–induced microcephaly in fetuses. Sci. Adv..

[19] Langerak T., Mumtaz N., Tolk V.I., Van Gorp E.C.M., Martina B.E., Rockx B., Koopmans M.P.G. (2019). The possible role of cross-reactive dengue virus antibodies in Zika virus pathogenesis. PLoS Pathog..

[20] Katzelnick L.C., Narvaez C., Arguello S., Mercado B.L., Collado D., Ampie O., Elizondo D., Miranda T., Carillo F.B., Mercado J.C. (2020). Zika virus infection enhances future risk of severe dengue disease. Science.

[21] Terzian A.C.B., Schanoski A.S., Mota M.T.D.O., Da Silva R.A., Estofolete C.F., Colombo T.E., Rahal P., Hanley K.A., Vasilakis N., Kalil J. (2017). Viral Load and Cytokine Response Profile Does Not Support Antibody-Dependent Enhancement in Dengue-Primed Zika Virus–Infected Patients. Clin. Infect. Dis..

[22] Davis D., Kaufmann R., Moticka E.J. (1998). Nonspecific immunity in pregnancy: Monocyte surface Fcgamma receptor expression and function. J. Reprod. Immunol..

[23] Jiang L., Sun Q. (2020). The Role of Autophagy-Mediated Dengue Virus Antibody-Dependent Enhancement Infection of THP-1 Cells. Intervirology.

[24] Dejnirattisai W., Jumnainsong A., Onsirisakul N., Fitton P., Vasanawathana S., Limpitikul W., Puttikhunt C., Edwards C., Duangchinda T., Supasa S. (2010). Cross-Reacting Antibodies Enhance Dengue Virus Infection in Humans. Science.

[25] Cui G., Si L., Wang Y., Zhou J., Yan H., Jiang L. (2020). Antibody-dependent enhancement (ADE) of dengue virus: Identification of the key amino acid that is vital in DENV vaccine research. J. Gene Med..

[26] Castanha P.M.S., Nascimento E.J.M., Braga C., Cordeiro M.T., de Carvalho O.V., de Mendonca L.R., Marques E.T. (2017). Dengue Virus-Specific Antibodies Enhance Brazilian Zika Virus Infection. J. Infect. Dis..

[27] Londono-Renteria B., Troupin A., Cardenas J.C., Hall A., Perez O.G., Cardenas L., Hartstone-Rose A., Halstead S.B., Colpitts T.M. (2017). A relevant in vitro human model for the study of Zika virus antibody-dependent enhancement. J. Gen. Virol..

[28] Kärber G. (1931). Beitrag zur kollektiven Behandlung pharmakologischer Reihenversuche. Naunyn-Schmiedebergs Arch. Für Exp. Pathol. Und Pharmakol..

[29] Michlmayr D., Andrade P., Gonzalez K., Balmaseda A., Harris E. (2017). CD14(+)CD16(+) monocytes are the main target of Zika virus infection in peripheral blood mononuclear cells in a paediatric study in Nicaragua. Nat. Microbiol..

[30] Stettler K., Beltramello M., Espinosa D.A., Graham V., Cassotta A., Bianchi S., Vanzetta F., Minola A., Jaconi S., Mele F. (2016). Specificity, cross-reactivity, and function of antibodies elicited by Zika virus infection. Science.

[31] Charles A.S., Christofferson R.C. (2016). Utility of a Dengue-Derived Monoclonal Antibody to Enhance Zika Infection In Vitro. PLoS Curr..

[32] Watanabe S., Tan N.W.W., Chan K.W.K., Vasudevan S. (2018). Dengue Virus and Zika Virus Serological Cross-reactivity and Their Impact on Pathogenesis in Mice. J. Infect. Dis..

[33] Li M., Zhao L., Zhang C., Wang X., Hong W., Sun J., Liu R., Yu L., Wang J., Zhang F. (2018). Dengue immune sera enhance Zika virus infection in human peripheral blood monocytes through Fc gamma receptors. PLoS ONE.

[34] Carlin A.F., Vizcarra E.A., Branche E., Viramontes K.M., Suarez-Amaran L., Ley K., Heinz S., Benner C., Shresta S., Glass C.K. (2018). Deconvolution of pro- and antiviral genomic responses in Zika virus-infected and bystander macrophages. Proc. Natl. Acad. Sci. USA.

[35] Hueston L., Ramirez R., Mahalingam S. (2017). Enhancement of Zika Infection by Dengue Virus–Specific Antibody Is Associated With Low Levels of Antiviral Factors. J. Infect. Dis..

[36] Chiofalo M.S., Teti G., Goust J.-M., Trifiletti R., La Via M.F. (1988). Subclass specificity of the Fc receptor for human IgG on K562. Cell. Immunol..

[37] Bournazos S., Wang T.T., Ravetch J.V. (2016). The Role and Function of Fcgamma Receptors on Myeloid Cells. Microbiol Spectr..

[38] Junker F., Gordon J., Qureshi O. (2020). Fc Gamma Receptors and Their Role in Antigen Uptake, Presentation, and T Cell Activation. Front. Immunol..

[39] Guilliams M., Bruhns P., Saeys Y., Hammad H., Lambrecht B.N. (2014). The function of Fcgamma receptors in dendritic cells and macrophages. Nat. Rev. Immunol..

[40] Bournazos S., Gupta A., Ravetch J.V. (2020). The role of IgG Fc receptors in antibody-dependent enhancement. Nat. Rev. Immunol..

[41] Rothman A.L. (2011). Immunity to dengue virus: A tale of original antigenic sin and tropical cytokine storms. Nat. Rev. Immunol..

[42] Foo S.-S., Chen W., Chan Y., Bowman J.W., Chang L.-C., Choi Y., Yoo J.S., Ge J., Cheng G., Bonnin A. (2017). Asian Zika virus strains target CD14(+) blood monocytes and induce M2-skewed immunosuppression during pregnancy. Nat. Microbiol..

[43] Paul L.M., Carlin E.R., Jenkins M.M., Tan A.L., Barcellona C.M., Nicholson C.O., Michael S.F., Isern S. (2016). Dengue virus antibodies enhance Zika virus infection. Clin. Transl. Immunol..

[44] Littaua R., Kurane I., Ennis F.A. (1990). Human IgG Fc receptor II mediates antibody-dependent enhancement of dengue virus infection. J. Immunol..

[45] Kontny U., Kurane I., Ennis F.A. (1988). Gamma interferon augments Fc gamma receptor-mediated dengue virus infection of human monocytic cells. J. Virol..

[46] Ubol S., Phuklia W., Kalayanarooj S., Modhiran N. (2010). Mechanisms of Immune Evasion Induced by a Complex of Dengue Virus and Preexisting Enhancing Antibodies. J. Infect. Dis..

[47] Boonnak K., Slike B.M., Burgess T.H., Mason R.M., Wu S.-J., Sun P., Porter K., Rudiman I.F., Yuwono D., Puthavathana P. (2008). Role of Dendritic Cells in Antibody-Dependent Enhancement of Dengue Virus Infection. J. Virol..

[48] Hamel R., Dejarnac O., Wichit S., Ekchariyawat P., Neyret A., Luplertlop N., Perera-Lecoin M., Surasombatpattana P., Talignani L., Thomas F. (2015). Biology of Zika Virus Infection in Human Skin Cells. J. Virol..

[49] Bilińska-Nigot B., Majkowski J. (1976). [Effect of diphenylhydantoin on a developed epileptogenic focus in cats with split cerebral hemispheres] Wplyw dwufenylohydantoiny (DPH) na rozwiniete ognisko padaczkowe u kotow z rozdzielonymi polkulami mozgowymi. Neurol Neurochir Pol..

[50] Yang D., Chu H., Lu G., Shuai H., Wang Y., Hou Y., Zhang X., Huang X., Hu B., Chai Y. (2021). STAT2-dependent restriction of Zika virus by human macrophages but not dendritic cells. Emerg Microbes Infect..

[51] Chen Y.C., Wang S.Y., King C.C. (1999). Bacterial lipopolysaccharide inhibits dengue virus infection of primary human monocytes/macrophages by blockade of virus entry via a CD14-dependent mechanism. J. Virol..

[52] Khaiboullina S., Uppal T., Sarkar R., Gorzalski A., Jeor S.S., Verma S.C. (2017). ZIKV infection regulates inflammasomes pathway for replication in monocytes. Sci. Rep..

[53] Strottmann D.M., Zanluca C., Mosimann A.L.P., Koishi A.C., Auwerter N.C., Faoro H., Cataneo A.H.D., Kuczera D., Wowk P.F., Bordignon J. (2019). Genetic and biological characterisation of Zika virus isolates from different Brazilian regions. Mem Inst Oswaldo Cruz..

[54] Bickel M. (1993). The role of interleukin-8 in inflammation and mechanisms of regulation. J. Periodontol..

[55] Zimmerman M.G., Quicke K.M., O’Neal J.T., Arora N., Machiah D., Priyamvada L., Kauffman R.C., Register E., Adekunle O., Swieboda D. (2018). Cross-Reactive Dengue Virus Antibodies Augment Zika Virus Infection of Human Placental Macrophages. Cell Host Microbe.

[56] Hemann E.A., Gale M., Savan R. (2017). Interferon Lambda Genetics and Biology in Regulation of Viral Control. Front Immunol..

[57] Narayan R., Tripathi S. (2020). Intrinsic ADE: The Dark Side of Antibody Dependent Enhancement During Dengue Infection. Front Cell Infect Microbiol..

[58] Martina B.E.E., Koraka P., Osterhaus A.D.M.E. (2009). Dengue Virus Pathogenesis: An Integrated View. Clin. Microbiol. Rev..

[59] Azeredo E.L., Zagne S.M., Santiago M.A., Gouvea A.S., Santana A.A., Neves-Souza P.C., Kubelka C.F. (2001). Characterisation of lymphocyte response and cytokine patterns in patients with dengue fever. Immunobiology.

[60] Langerak T., Broekhuizen M., Unger P.-P.A., Tan L., Koopmans M., van Gorp E., Danser A.H.J., Rockx B. (2022). Transplacental Zika virus transmission in ex vivo perfused human placentas. PLoS Neglected Trop. Dis..

[61] Vidarsson G., Dekkers G., Rispens T. (2014). IgG Subclasses and Allotypes: From Structure to Effector Functions. Front. Immunol..

[62] Dekkers G., Treffers L., Plomp R., Bentlage A.E.H., De Boer M., Koeleman C.A.M., Lissenberg-Thunnissen S.N., Visser R., Brouwer M., Mok J.Y. (2017). Decoding the Human Immunoglobulin G-Glycan Repertoire Reveals a Spectrum of Fc-Receptor- and Complement-Mediated-Effector Activities. Front. Immunol..

[63] Hayes J.M., Wormald M.R., Rudd P.M., Davey G.P. (2016). Fc gamma receptors: Glycobiology and therapeutic prospects. J. Inflamm. Res..

[64] Crooks C.M., Weiler A.M., Rybarczyk S.L., Bliss M.I., Jaeger A.S., Murphy M.E., Simmons H.A., Mejia A., Fritsch M.K., Hayes J.M. (2021). Previous exposure to dengue virus is associated with increased Zika virus burden at the maternal-fetal interface in rhesus macaques. PLoS Neglected Trop. Dis..

[65] Zimmerman M.G., Wrammert J., Suthar M.S. (2020). Cross-Reactive Antibodies during Zika Virus Infection: Protection, Pathogenesis, and Placental Seeding. Cell Host Microbe.

