# Supplementary material for: Comparative Analysis of In Vitro Models to Study Antibody-Dependent Enhancement of Zika Virus Infection
Source: Viruses. 2022 Dec 13;14(12):2776. doi: 10.3390/v14122776 (PMC9781448; doi:10.3390/v14122776)
Supplement: Supplementary file 1 [file viruses-14-02776-s001.zip › viruses-2057486-supplementary.pdf]

Supplementary figures

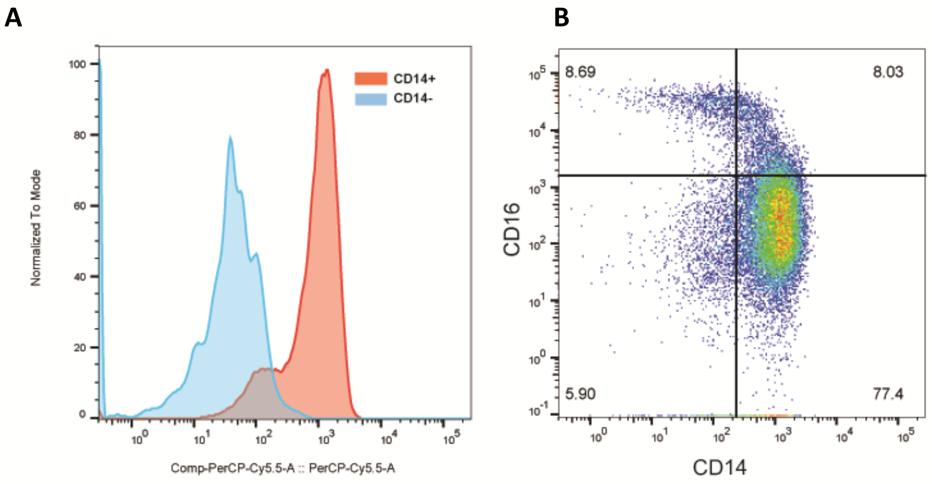

Figure S1: CD14+ expression of monocytes that were magnetically isolated from frozen PBMCs using anti-human CD14 microbeads

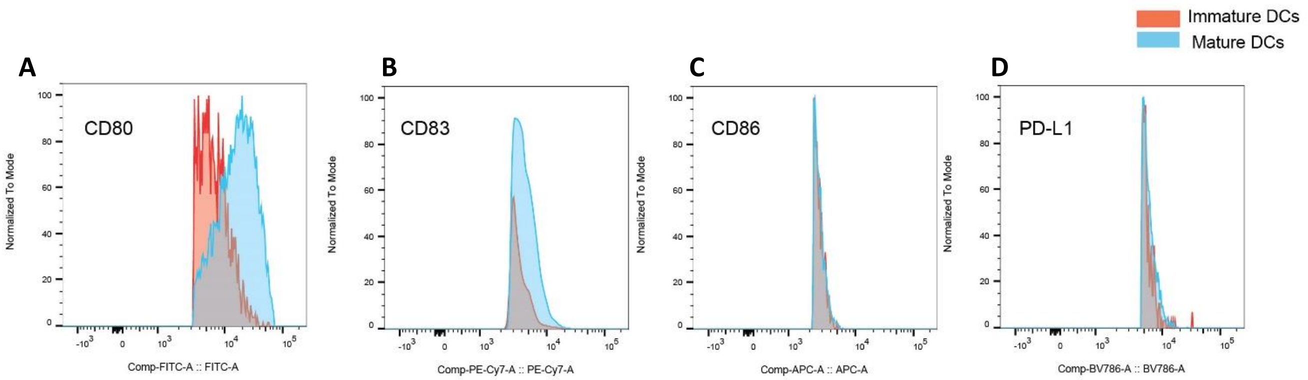

Figure S2: Characterization of dendritic cell surface expression markers using flow cytometry.

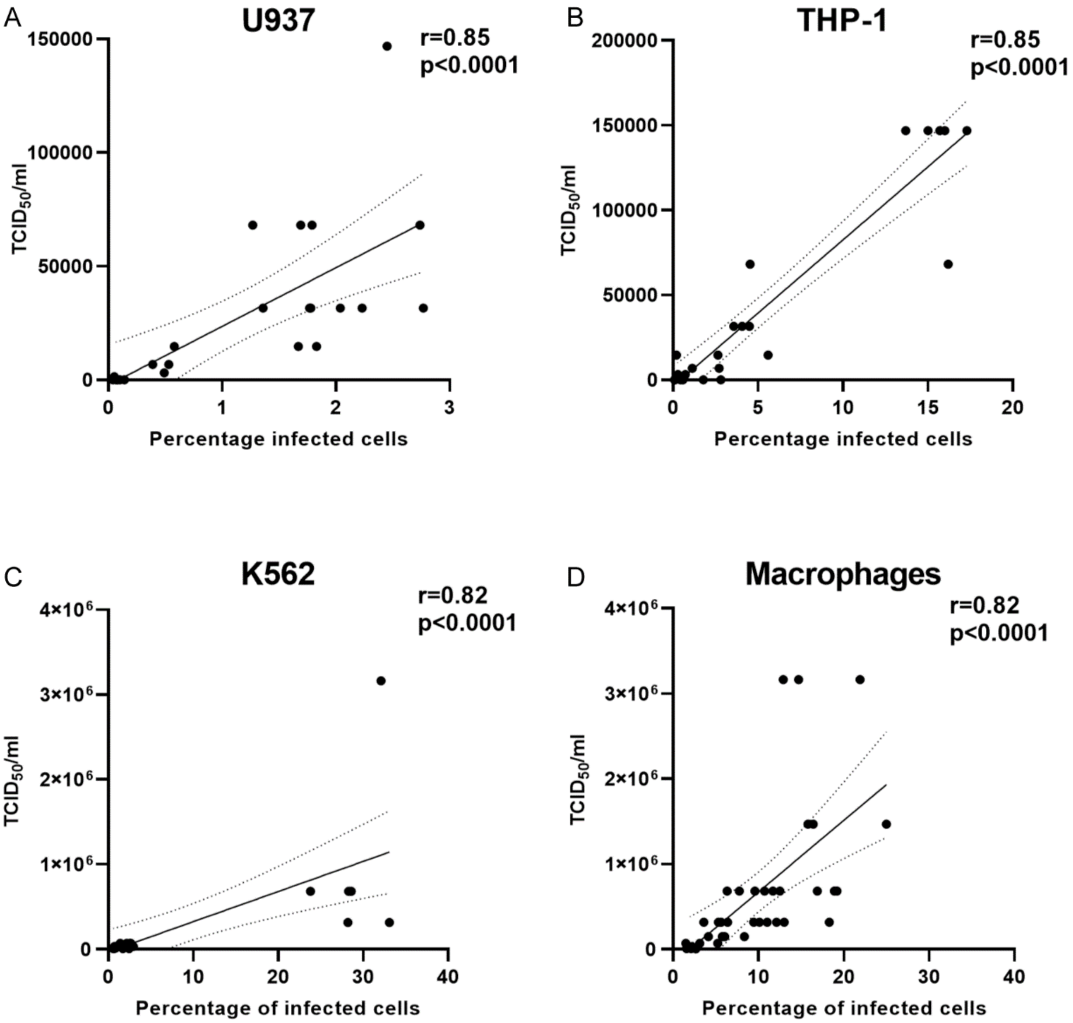

Figure S3: Strong correlation between viral titers in supernatants and percentage of infected cells U937 cells

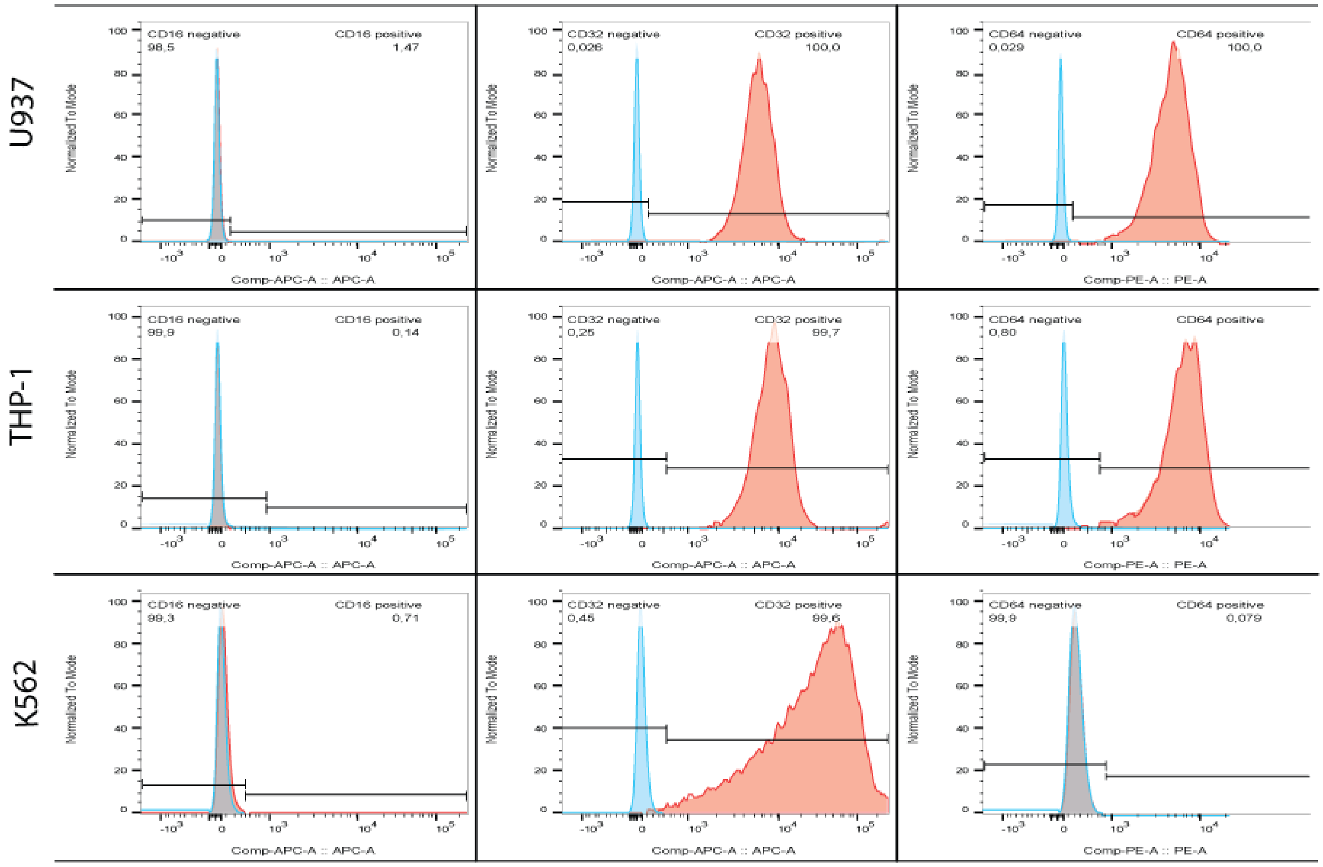

Figure S4: FcγR expression in U937, THP-1 and K562 cells. Cells are illustrated in the red graphs while isotype controls are illustrated in blue.

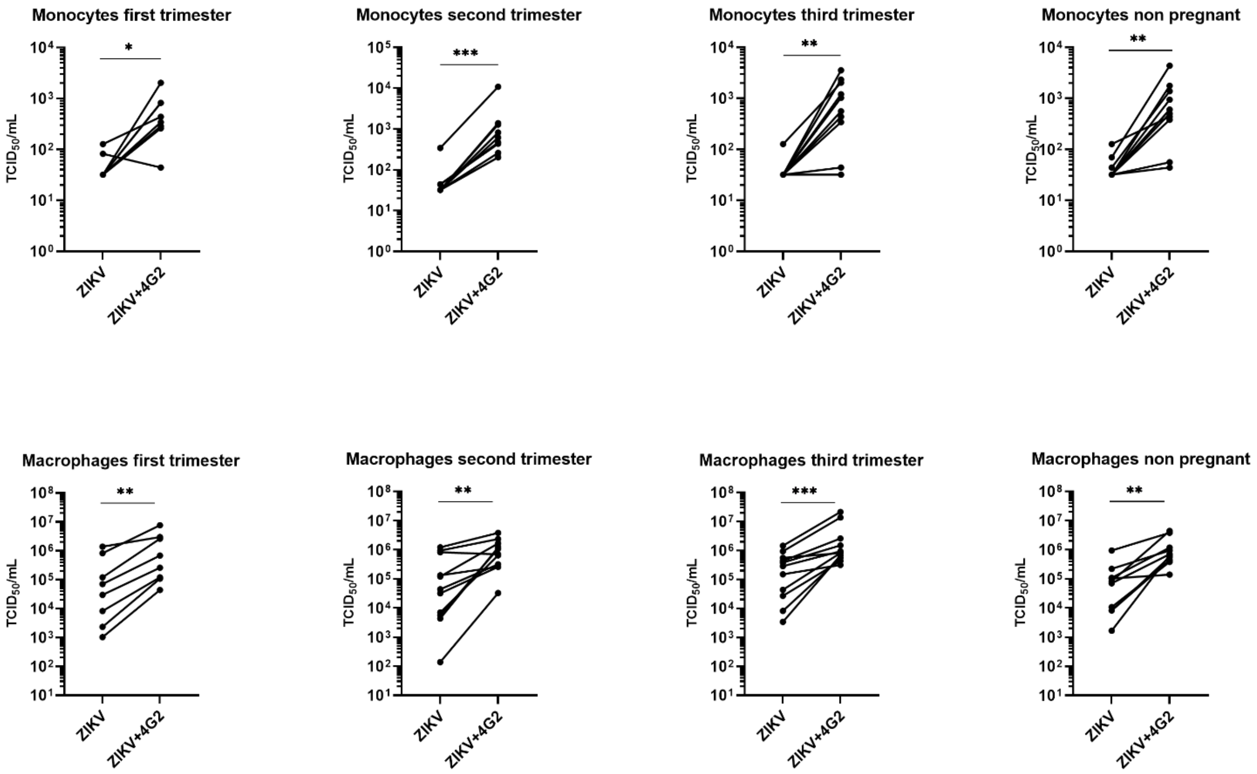

Figure S5: Paired ZIKV titers determined in supernatants of monocytes and macrophages from pregnant women (per trimester) and non-pregnant women infected with ZIKV and with ZIKV+hu4G2 to induce ADE of infection. Statistical analysis was performed with a paired Mann-Whitney U test.

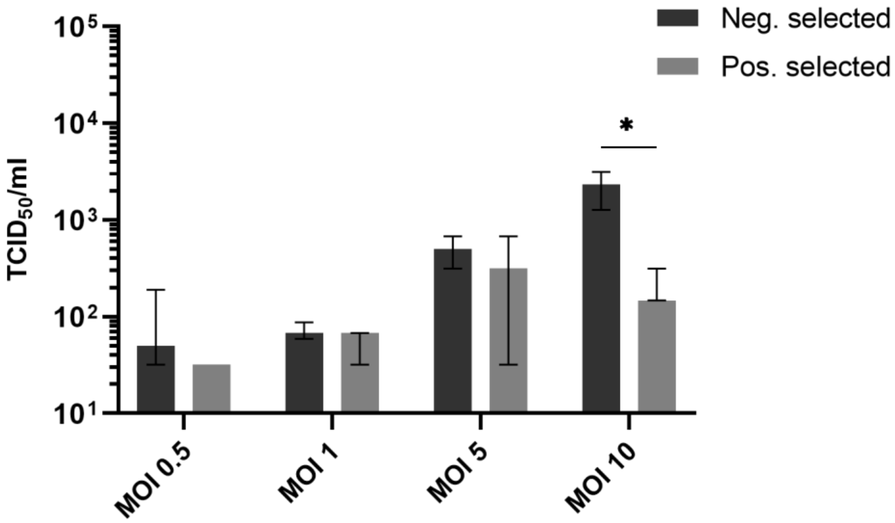

Figure S6: Monocytes were isolated from PBMC's with a CD14+ isolation kit (pos. selected) or with a pan monocyte isolation kit in which monocytes are negatively selected (untouched, neg. selected).
